# In Situ Formation of Surface-Induced Oxygen Vacancies in Co_9_S_8_/CoO/NC as a Bifunctional Electrocatalyst for Improved Oxygen and Hydrogen Evolution Reactions

**DOI:** 10.3390/nano11092237

**Published:** 2021-08-30

**Authors:** Khalil ur Rehman, Shaista Airam, Xiangyun Lin, Jian Gao, Qiang Guo, Zhipan Zhang

**Affiliations:** Key Laboratory of Photoelectronic/Electrophotonic Conversion Materials, Key Laboratory of Cluster Science, Ministry of Education of China, School of Chemistry and Chemical Engineering, Beijing Institute of Technology, Beijing 100081, China; meer@bit.edu.cn (K.u.R.); shaistaerum25@yahoo.com (S.A.); lin_xiangyun@foxmail.com (X.L.); gaojian8908@163.com (J.G.); guoqiang15124780173@gmail.com (Q.G.)

**Keywords:** oxygen vacancies, heterojunction, oxygen evolution reaction, hydrogen evolution reaction

## Abstract

Creating oxygen vacancies and introducing heterostructures are two widely used strategies in Co-based oxides for their efficient electrocatalytic performance, yet both strategies have rarely been used together to design a bifunctional electrocatalyst for an efficient overall water splitting. Herein, we propose a facile strategy to synthesize oxygen-defect-rich Co_9_S_8_/CoO hetero-nanoparticles with a nitrogen-doped carbon shell (ODR-Co_9_S_8_/CoO/NC) through the in situ conversion of heterojunction along with surface-induced oxygen vacancies, simply via annealing the precursor Co_3_S_4_/Co(OH)_2_/ZIF-67. The as-prepared ODR-Co_9_S_8_/CoO/NC shows excellent bifunctional catalytic activities, featuring a low overpotential of 217 mV at 10 mA cm^−2^ in the oxygen evolution reaction (OER) and 160 mV at 10 mA cm^−2^ in the hydrogen evolution reaction (HER). This performance excellency is attributed to unique heterostructure and oxygen defects in Co_9_S_8_/CoO nanoparticles, the current work is expected to offer new insights to the design of cost-effective, noble-metal-free electrocatalysts.

## 1. Introduction

Concerns on fossil fuel reserves and environmental issues have urged scientists to explore renewable energy reservoirs to find substitutes to traditional fossil fuels [1,2,3,4,5]. In particular, electrochemical water splitting into hydrogen and oxygen via the hydrogen evolution reaction (HER) and oxygen evolution reaction (OER) is one of the most attractive options [6,7]. Literally, it requires electrocatalysts to diminish the overpotentials in the OER and HER to maximize the conversion efficiency [8,9]. Traditionally, electrocatalysts based on noble metals have been predominantly used in these tasks (e.g., IrO_2_ and RuO_2_ in the OER and Pt in the HER) [10], yet their costs and scarcities significantly limit their industrial applications. So, the development of highly active noble metal-free electrocatalysts is of exceptional importance [11,12]. Lately, as efficient electrocatalysts, cobalt oxides (Co_3_O_4_ and CoO) have drawn tremendous attraction due to distinctive features such as their 3D electronic structure, feasible synthesizing methodologies, and efficient catalytic activity [13,14,15,16,17,18,19,20,21]. In spite of this distinctiveness, their poor intrinsic electronic conductivity and inferior bifunctionality for overall electrochemical water splitting have hindered their practical applications [22,23]. To resolve its intrinsic conductivity issue, it was reported while creating oxygen vacancies in these oxides has altered the electronic environment that acted critically to induce conductivity [24,25,26]. Xu et al. has fabricated plasma-engraved oxygen vaccines in Co_3_O_4_ nanosheets and reported its improved OER performance (*η* of 300 mV at *J*_OER_ = 10 mA cm^−2^) over pristine Co_3_O_4_ nanosheets (*η* of 540 mV at *J*_OER_ = 10 mA cm^−2^). Oxygen defects in plasma-engraved Co_3_O_4_ nanosheets were noticed to induce enhancement of Co^2+^ population with distinctively exposed active sites and highly increased surface area which has improved its electrical conductance [27]. Liang et al. synthesized CoO hexagrams having numerous oxygen defects and they demonstrated an *η* of ~260 mV at *J*_OER_ = 10 mA cm^−2^. Moreover, theoretical explanations revealed this performance excellency was attributed to the abundant oxygen defects in CoO that lowered the activation energy barrier and improved the electrical conductivity as well [28]. However, for electrochemical bifunctional catalysis, fabrication of cobalt-based oxides with HER active counterpart could develop a heterojunction that has manipulated distinctive features for bifunctionality [29]. DFT and experimental studies have supported the potential role of heterojunction to enhance the catalytic conductivity for electrochemical water splitting purposes due to an altered electronic environment exposing more active sites with efficient chemisorption properties [30,31,32,33]. For example, Muthurasu et al. synthesized a Co_3_O_4_/MoS_2_ heterostructure, and the synergic effects between MoS_2_ and Co_3_O_4_ enabled the catalyst an efficient OER as well as HER with *η* values of 230 mV (*J*_OER_ = 20 mA cm^−2^) and 205 mV (*J*_HER_ = 10 mA cm^−2^) respectively. Interfacial coupling in Co_3_O_4_/MoS_2_ heterostructure has enhanced the binding affinities for oxygen and hydrogen-carrying intermediates, which in turn improved overall water splitting [34]. Peng et al. synthesized Co_9_S_8_/Co_3_O_4_ nano-heterostructure which manifested an OER *η* of 250 mV and HER *η* of 360 mV at 10 mA cm^−2^ as well. The heterostructure formation created such structural alignments which lowered the activation energy barrier, enhanced the absorption of intermediates, and also accelerated the overall electrochemical splitting [29]. Generally, the generation of carbon-matrix-woven heterostructures needed a lengthy/intricate synthetic procedure, while metal-organic frameworks (MOFs) can easily produce metal sulfides and/or metal oxides heterostructures along with carbon frameworks [35,36,37]. The inherited frame structure of MOF precursors along with embedded heterostructured nanoparticles in graphitic carbon framework remarkably improves the electrocatalytic performance and the stability of the catalyst [38].

Therefore, it is believed that structural designing in cobalt-based oxides may prove more effective, such as by creating oxygen vacancies and interfacial coupling may fabricate an efficient and highly conductive bifunctional electrocatalyst for water splitting purposes.

Moreover, doping the carbon framework with heteroatoms, such as N with stronger electronegativity, is highly attractive, as this induces carbon atoms to serve as accessible active sites in catalysis by promoting a positive charge density on them [39,40]. In the hybrid structure of nanoparticles embedded in N-doped carbon, nanoparticles can be isolated by covered carbon materials to fully expose their catalytic sites and the carbon framework protects inner nanoparticles from unwanted side reactions for better long-term stability [40,41,42].

Herein, we have adopted a facile strategy to synthesize ODR-Co_9_S_8_/CoO/NC by in situ generation of heterojunction and surface-induced oxygen vacancies via annealing well-dispersed Co_3_S_4_ in the ZIF-67 framework. Benefiting from these distinctive features, the as-prepared catalyst shows an *η* of 217 mV (*J*_OER_ = 10 mA cm^−2^) in the OER and an *η* of 160 mV (*J*_HER_ = 10 mA cm^−2^) in the HER, qualifying it as the best OER/HER bifunctional catalyst among reported Co-based compounds. The following factors are accountable for improved bifunctionality of the ODR-Co_9_S_8_/CoO/NC: (i) the generation of oxygen vacancies produces more active defects for OER and also alters the surface electronic structure to enhance the electrocatalytic activity; (ii) the formation of heterostructure offers many active sites for optimization of adsorption as well as desorption free energies of reactants/intermediates to accelerate the sluggish step with ultimate fast water dissociation in alkaline electrolytes; (iii) the carbon framework along with N-doping, protects the hetero-nanoparticles from catalytic corrosion to ensure catalytic stability. The current work is expected to provide new insights into the designing and synthesis of new noble-metal-free bifunctional electrocatalysts with improved OER and HER activities. 

## 2. Experimental Section

### 2.1. Chemicals

Co(NO_3_)_2_·6H_2_O (98.0%), C_2_H_5_NS (99%), C_4_H_6_N_2_ (98%) and C_2_H_6_O_2_ (98%) (Aladdin, Shanghai, China), commercial RuO_2_ (99.9%) and Pt/C (99.9%) (Sigma-Aldrich, Shanghai, China). Where Co(NO_3_)_2_.6H_2_O and C_4_H_6_N_2_ have been used as ZIF-67 precursors. All above-mentioned chemical reagents were used without any further refinement. For overall experimentations, distilled water has been used.

### 2.2. Method

For the synthesis of ODR-Co_9_S_8_/CoO/NC heterostructure nanocomposite, firstly, ZIF-67 was prepared, for which 0.06 M cobalt (II) nitrate hexahydrates solution (50 mL) was gradually added into 2.16 M 2-methylimidazole solution (50 mL) and ultrasonically stirred for 30 min at room temperature. Then, 30 mL of 0.13 M thioacetamide solution (10 mL distilled water and 20 mL ethylene glycol) was poured into the above ZIF-67 solution with subsequent 1 h vigorous stirring at 25 ℃ and then shifted into an autoclave (100 mL) where it was solvothermally treated for 18 h at 180 °C. After the autoclave was cooled, the as-formed product was centrifuged and rinsed repeatedly to obtain the Co_3_S_4_/Co(OH)_2_/ZIF-67 precursor. Solvothermally grown precursor (Co_3_S_4_/Co(OH)_2_/ZIF-67) was then calcined for 3 h at 650 °C under inert conditions to synthesize the oxygen defect-rich Co_9_S_8_/CoO/NC heterostructure. For comparison, the CoO/NC composite was synthesized by following the reported method [43]. Generally, it is prepared by annealing ZIF-67 for 5 min at 530 °C under Ar flow. Pure Co_9_S_8_ was also obtained through the same process without adding 2-methylimidazole. 

### 2.3. Material Characterization

The scanning electron microscopy (SEM) and transmission electron microscopy (TEM) measurements were performed with JSM-7001F (Tokyo, Japan) and JEM 2100 (Tokyo, Japan) electron microscopes, while for X-ray diffraction (XRD), 1710 diffractometer (Netherland) was used to record data. For X-ray photoelectron spectra (XPS), ESCALab220i-XL electron spectrometer (VG Scientific Waltham, MA, USA) was used for material characterization. For a description of XPS spectra deconvolution, Shirley and Linear function fitted background was applied. While for data interpretation, XPS peak software was used.

### 2.4. Electrochemical Measurements

A conventional three electrodes set up in the presence of basic media (0.1 M KOH solution) has been utilized to pursue electrochemical measurements on CHI 760D electrochemical workstation. The voltage of Ag/AgCl was calibrated to RHE as shown in the given condition:

E_RHE_ = E_Ag/AgCl_ + 0.197 + 0.059 pH (1)

To make catalyst ink, 5 mg sample has added in 1000 μL of the solvent mixture containing 100 μL of 5% Nafion solution, 450 μL ethanol, and 450 μL DI water and then loaded on pre-polished GCE (3 mm diameter) through dripping the 4 μL catalyst ink (≈0.283 mg cm^−2^). For minimizing the double-layer charging current, linear sweep voltammetric (LSV) tests have been performed with 5 mV s ^−1^ scanning speed. All the LSV data were 100% compensated to remove the ohmic voltage. For the stability test, chronopotentiometric tests (i-t) were performed at 20 mA cm^−2^. From the LSV curves, a Tafel plot has been assessed by employing the given relation.

η = a + b log j
(2)

Overpotential for HER and OER was determined by utilizing the following equation:

η = 0 − E_RHE_ (3)

η = E_RHE_ − 1.23 (4)

EIS measurements were performed at 10^5^ to 0.1 Hz frequency range under applied voltage equivalent to the potential at *j* ~ 10 mA cm^−2^. 

## 3. Result and Discussion

**Figure 1:**
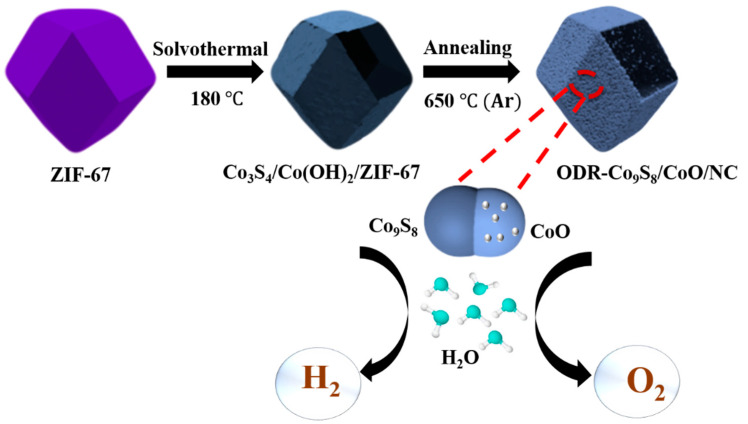
Schematic diagram of ODR-Co_9_S_8_/CoO/NC.

The ODR-Co_9_S_8_/CoO/NC heterostructure was prepared by a facile two-step methodology presented in Figure 1. The zeolite imidazole framework-67 (ZIF-67) with the characteristic crystal structure and dodecahedral rhombic morphology (Figures S1a,b, S2a and S3) were used as the cobalt source. In the presence of thioacetamide, the solvothermal treatment of ZIF-67 led to the in situ formation of Co_3_S_4_/Co(OH)_2_/ZIF-67 [44,45]. The coexistence of both cobalt sulfide (Co_3_S_4_) and cobalt hydroxide Co(OH)_2_ in the resulted precursor was evidenced by XRD, FTIR, EDS, and XPS (Figures S2a,b, S4 and S5). During annealing under an Ar atmosphere at 650 °C, thermal decomposition of Co(OH)_2_ into CoO and phase transition of Co_3_S_4_ into Co_9_S_8_ resulted in the conversion of Co_3_S_4_/Co(OH)_2_/ZIF-67 to ODR-Co_9_S_8_/CoO/NC heterostructure [28].

**Figure 2:**
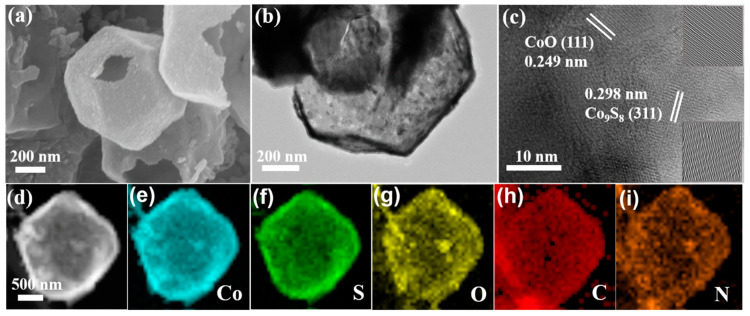
SEM, TEM, HRTEM images (**a**–**c**) and elemental distributions (**d**–**i**) of the ODR- Co_9_S_8_/CoO/NC heterostructure.

SEM was used initially for the exploration of the morphology and microstructure of the ODR-Co_9_S_8_/CoO/NC heterostructure. As shown in Figure 2a, ODR-Co_9_S_8_/CoO/NC retained the uniform polyhedral structure inherited from the precursor (ZIF-67), while the cracks confirmed its hollow nature with shells of 20–30 nm in thickness. TEM images further revealed that the hollow polyhedral structure was consist of nanoparticles with an outermost carbon layer (Figure 2b). In Figure 2c, more speculation by HRTEM has declared that these nanoparticles were composed of both Co_9_S_8_ and CoO (hetero particles). Moreover, lattice fringes profiles (Figure S6) were conferred with 0.298 nm and 0.249 nm interplanar spacing that were assigned to (311) and (111) planes in Co_9_S_8_ and CoO, respectively [46,47]. Energy-dispersive X-ray analysis (EDX) (Figure S7) and energy-dispersive elemental mapping results (Figure 2d–i) further proved the coexistence and homogeneous distribution of Co, S, O, and N elements in the carbon matrix.

**Figure 3:**
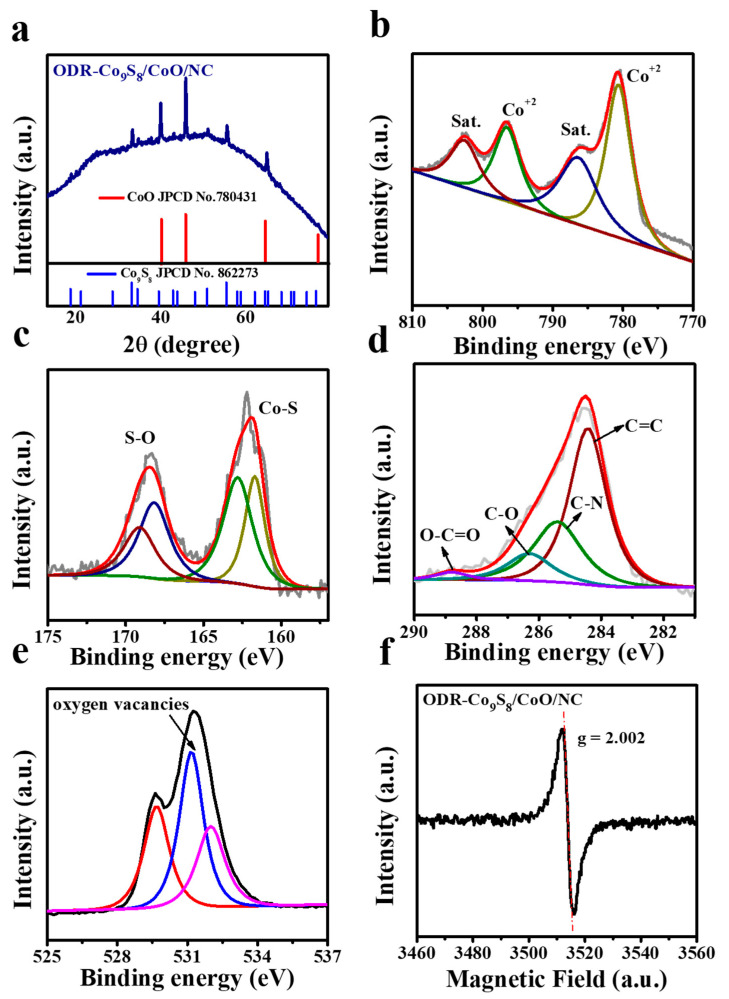
(**a**) XRD signals for ODR-Co_9_S_8_/CoO/NC heterostructures; (**b**–**e**) Core-level spectra of Co 2p, S 2p, C 1s, and O 1s elements of ODR-Co_9_S_8_/CoO/NC heterostructures; (**f**) EPR for ODR-Co_9_S_8_/CoO/NC.

The crystalline structure of the ODR-Co_9_S_8_/CoO/NC heterostructure was characterized by the powder XRD pattern (Figure 3a). The as-prepared ODR-Co_9_S_8_/CoO/NC heterostructure exhibited two types of characteristic diffraction peaks. The peaks at 2 theta of 36.6°, 42.6°, 61.5°, and 73.7° corresponded to (111), (200), (220), and (311) planes of cubic CoO (JCPDS No. 78-431), [45] which is due to the thermal decomposition of Co(OH)_2_ to CoO at high temperature under an Ar atmosphere [48]. While the set of peaks at 15.5°, 17.8°, 25.1°, 29.5°, 31.1°, 39.4°, 44.7°, 47.6°, and 52.3° can be ascribed to (111), (200), (220), (311), (222), (331), (422), (511) and (440) planes of cubic Co_9_S_8_ (JPCD NO. 86-2273) [49]. Quantitative surface elemental composition and chemical states of ODR-Co_9_S_8_/CoO/NC heterostructure were probed by XPS in Figure 3b–e, where Co 2p, S 2p, C 1s, and O 1s deconvolutions have been described and atomic ratios details are enlisted in Table S1. For Co 2p spectrum (Figure 3b), the peaks at 780.6 eV and 796.5 eV were assigned to the binding energy of Co 2p_3/2_ and Co 2p_1/2_ levels of Co_9_S_8_ and CoO, respectively, while two satellite peaks at 786.1 and 802.8 eV implied the presence of Co^2+^ in the heterostructure in good consistency with the published literature [49,50]. While the absence of Co^3+^ peaks in Co2p spectrum of ODR-Co_9_S_8_/CoO/NC heterostructure is possibly due to the efficient thermal conversion of the intermediate species (Co(OH)_2_/Co_3_O_4_) into CoO to form pure phase heterostructure with the appearance of Co^2+^ sharp peaks for Co2p spectrum [50,51,52,53]. For the S 2p spectrum (Figure 3c), the doublet for S 2p_3/2_ and S 2p_1/2_ at 161.2 eV and 162.5 eV originated from sulfur atoms in Co_9_S_8_ [54]. Additionally, peaks at 168.2 eV and 169.1 eV were attributed to the existence of O=S=O and SO_4_ bonds, suggesting a chemical coupling between Co_9_S_8_ and CoO [55]. The peaks at 286.3 eV and 288.8 eV in the C 1s spectrum were related to the presence of C–O and O–C=O bonds while the other two peaks at 284.5 and 285.4 have indicated that (Figure 3d) graphitic carbon (C=C) and N-dopped carbon (C–N) are dominant in the heterostructure and believed to play a vital role in improving the heterostructure conductivity by facilitating the charge transportation [43,48]. For O 1s spectrum (Figure 3e), three peaks at 529.5 eV, 531.2 eV, and 532 eV were ascribed to the lattice oxygen, [56] numerous defects with lower coordination number for oxygen [57,58] and the presence of hydroxyl group [58] in the ODR-Co_9_S_8_/CoO/NC heterostructure, respectively. The significant presence of oxygen defects in the ODR-Co_9_S_8_/CoO/NC heterostructure is considered pivotal to boost the performance of OER [59]. The electron paramagnetic resonance (EPR) analysis was conducted to affirm the oxygen vacancies presence in ODR-Co_9_S_8_/CoO/NC heterostructure. As depicted in Figure 3f, a peak with a g factor of 2.002 could be attributed to oxygen vacancies on the surface of the ODR-Co_9_S_8_/CoO/NC heterostructure [60].

**Figure 4:**
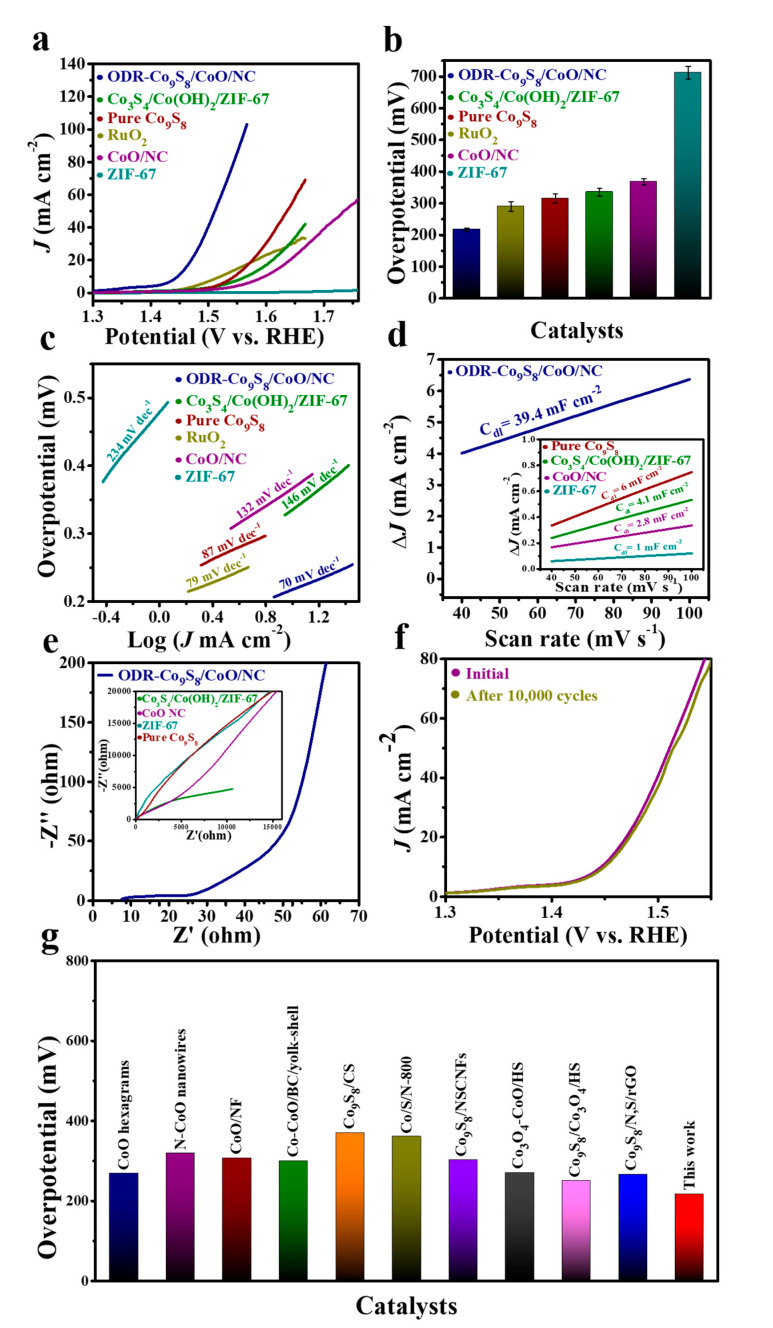
(**a**) OER performance (**b**) Overpotential comparison (The error bar represents the range of results from three independent measurements) and (**c**) Tafel slope of ODR-Co_9_S_8_/CoO/NC, RuO_2_, Co_3_S_4_/Co(OH)_2_/ZIF-67, ZIF-67, Co_9_S_8_, and CoO/NC in 0.1 M KOH. (**d**) Capacitive current measurements (ΔJ_0_ = J_a_ − J_c_) and (**e**) Nyquist plots for ODR-Co_9_S_8_/CoO/NC, Co_3_S_4_/Co(OH)_2_/ZIF-67, ZIF-67, Co_9_S_8_, and CoO/NC. (**f**) The polarization curves after the first and 10,000th CV cycles. (**g**) Overpotential comparison of the as-prepared catalyst with previously reported Co-based compounds at *J_OER =_* 10 mA cm^−2^.

The OER activity of the ODR-Co_9_S_8_/CoO/NC heterostructure was electrochemically accessed in a conventional three-electrode system in 0.1 M KOH solution. For control samples, under the same conditions, the OER performances of ZIF-67, Co_3_S_4_/Co(OH)_2_/ZIF-67, CoO/NC, pure Co_9_S_8_, and commercial RuO_2_ were also taken. Figure 4a shows that the ODR-Co_9_S_8_/CoO/NC heterostructure exhibited superior OER performance to other reference samples and *η* values (at *J*_OER_ = 10 mA cm^−2^) of all catalysts are summarized in Figure 4b. The improvement in the catalytic activity of the ODR-Co_9_S_8_/CoO/NC in OER can be rationalized by rapid charge transfer and water dissociation induced by the generation of oxygen defects and the in situ formation of heterojunction [27,61]. Theoretical results have shown that the introduction of oxygen vacancies and heterojunction modulates the surface electronic structure of the catalysts by inducing denser electron density around the Fermi level, [62,63] where oxygen vacancies tend to generate inter-band states for improved conductivity and the formation of heterojunction remodels the d-band center to circumvent unwanted charge transfer resistance for fast reaction kinetics. Clearly, the ODR-Co_9_S_8_/CoO/NC heterostructure possessed the lowest *η* (217 mV), as compared to those of ZIF-67 (712 mV), Co_3_S_4_/Co(OH)_2_/ZIF-67 (335 mV), CoO/NC (367 mV), pure Co_9_S_8_ (315 mV) and RuO_2_/C (290 mV). The kinetics of the catalyzed OER was studied by Tafel slopes using the polarization curves (Figure 4c) [64]. The ODR-Co_9_S_8_/CoO/NC heterostructure showed a Tafel slope value of 70 mV dec ^−1^, distinguishably lowest among ZIF-67 (234 mV dec^−1^), Co_3_S_4_/Co(OH)_2_/ZIF-67 (146 mV dec^−1^), CoO/NC (132 mV dec^−1^), pure Co_9_S_8_ (87 mV dec^−1^) and RuO_2_/C (79 mV dec^−1^). This lowest Tafel slope value (70 mV dec^−1)^ of ODR-Co_9_S_8_/CoO/NC heterostructure suggested that one-electron equilibrium proceeds a chemical rate-limiting step in OER [65]. In addition, at the electrode/electrolyte interface, the ECSA of different reference catalysts was determined from their C_dl_ values which were calculated from CV curves in Figure S8a–c. As depicted in Figure 4d and Table S2, the ODR-Co_9_S_8_/CoO/NC possessed a greater C_dl_ value (39.4 mF cm^−2^) than those of pure Co_9_S_8_ (6 mF cm^−2^), Co_3_S_4_/Co(OH)_2_/ZIF-67 (4.1 mF cm^−2^), CoO/NC (2.8 mF cm^−2^) and ZIF-67 (1 mF cm^−2^) suggesting numerous exposed active sites in the OER reaction. Moreover, ECSA normalized linear sweep voltammetry (LSV) curves (Figure S9a) of the ODR-Co_9_S_8_/CoO/NC demonstrating superior OER performance to ZIF-67, Co_3_S_4_/Co(OH)_2_/ZIF-67, CoO/NC, pure Co_9_S_8_, and RuO_2_/C which further indicates that the enhancement in intrinsic activity is due to the improved conductivity due to oxygen vacancies in ODR-Co_9_S_8_/CoO/NC heterostructure.

Furthermore, the mass activity (1.285 A mg^−1^) and TOF (9.3 × 10 ^−3^ mol s^−1^) in ODR-Co_9_S_8_/CoO/NC is superior to individual samples (Figure S10a and Table S3). In the EIS spectra of Figure 4e, the ODR-Co_9_S_8_/ CoO/NC heterostructure exhibited a significantly reduced semicircle, further confirming its rapid charge transfer kinetics due to the formation of heterojunction in the ODR-Co_9_S_8_/CoO/NC heterostructure for efficient OER. The polarization curve in Figure 4f illustrated that the ODR-Co_9_S_8_/CoO/NC heterostructure barely showed degradation even after 10,000 CV cycles validating it as a stable and durable electrocatalyst in the alkaline medium for the OER. Additionally, The ODR-Co_9_S_8_/CoO/NC showed similar OER polarization curves even at different scan rates (1 to 100 mV s^−1^) as depicted in (Figure S11a) which indicates its stability for the active electrochemical process in alkaline solution. The crystalline phase of ODR-Co_9_S_8_/CoO/NC after the stability test was also determined. As depicted in Figure S12 the crystalline structure of the catalyst was intact. This structural solidity may be attributed to the protection from the carbon framework that helped electrocatalytic active species to resist degradation even under severe conditions (strong alkaline conditions) after long-term stability tests [40,42]. One concern for the dependency of the catalytic performance on oxygen vacancies needed speculation, for that, control samples of Co_9_S_8_/CoO/NC heterostructure were annealed at 450 °C and 650 ℃ before electrochemical measurements in Figure S13. Where the EPR spectra of the sample annealed at 650 ℃ possessed strong signals with g = 2.002 confirming the formation of more concentration of oxygen vacancies than the sample annealed at 450 ℃. As a result, the sample annealed at 650 ℃ showed much higher catalytic current densities than that annealed at 450 ℃, suggesting dependency of catalytic performance of the catalyst on oxygen vacancies [28]. Additionally, performance-based comparative exploration in-relation to already outlined cobalt-metal-based oxides as well as sulfides, ODR-Co_9_S_8_/CoO/NC heterostructure exhibited conspicuously worth-noticing reduced *η* values for OER (Table S4 of Supporting Information and Figure 4g).

**Figure 5:**
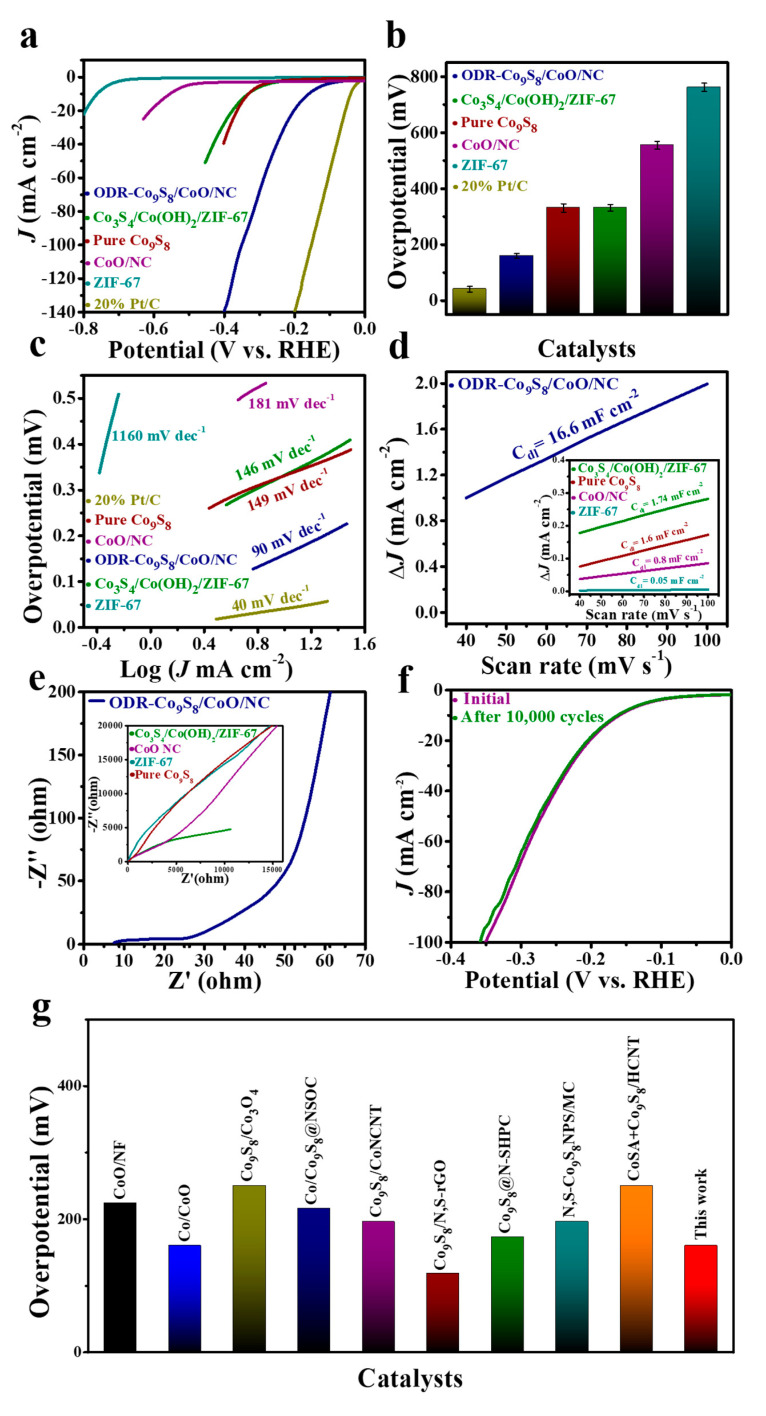
(**a**) HER performance, (**b**) Overpotential comparison (The error bar represents the range of results from three independent measurements). (**c**) Tafel slope of ODR-Co_9_S_8_/CoO/NC, 20% Pt/C, Co_3_S_4_/Co(OH)_2_/ZIF-67, ZIF-67, Co_9_S_8_, and CoO/NC in 0.1 M KOH. (**d**) Capacitive current measurements (ΔJ_0_ = J_a_ − J_c_) and (**e**) Nyquist plots for ODR-Co_9_S_8_/CoO/NC, Co_3_S_4_/Co(OH)_2_/ZIF-67, ZIF-67, Co_9_S_8_ and CoO/NC. (**f**) Durability of ODR-Co_9_S_8_/CoO/NC electrocatalyst after first and 10,000th CV cycles. (**g**) Overpotential comparison of the as-prepared catalyst with previously reported Co-based compounds at *J*_HER_ = 10 mA cm^−2^.

The ODR-Co_9_S_8_/CoO/NC can also work as an efficient electrocatalyst in the HER. Figure 5a compares linear sweep voltammetry (LSV) curves of the ODR-Co_9_S_8_/CoO/NC, ZIF-67, Co_3_S_4_/Co(OH)_2_/ZIF-67, CoO/NC, pure Co_9_S_8_, and commercial Pt/C. Unsurprisingly, Pt/C showed the lowest *η* of 41 mV at *J_HER_* = 10 mA cm^−2^, yet the ODR-Co_9_S_8_/CoO/NC heterostructure featured a second-lowest *η* of 160 mV that is significantly lower than those of ZIF-67 (762 mV), Co_3_S_4_/Co(OH)_2_/ZIF-67 (332 mV), CoO/NC (555 mV) and pure Co_9_S_8_ (331 mV) (Figure 5b). This performance excellency again related to the formation of oxygen vacancies and heterojunction in the ODR-Co_9_S_8_/CoO/NC catalyst with prompt properties of efficient water dissociation and rapid charge transfer for better HER performance [27,61]. Meanwhile, the ODR-Co_9_S_8_/CoO/NC heterostructure showed a 90 mV dec^−1^ Tafel slope (Figure 5c) and this value was considerably smaller than those of ZIF-67, Co_3_S_4_/Co(OH)_2_/ZIF-67, CoO/NC, and pure Co_9_S_8_ (1160 mV dec^−1^, 146 mV dec^−1^, 181 mV dec^−1^, 149 mV dec^−1^, respectively), suggesting a Volmer–Heyrovsky mechanism for the HER on the surface of the ODR-Co_9_S_8_/CoO/NC [66]. As plotted in Figure 5d and Table S2, the C_dl_ value calculated from CV curves (Figure S8d) for the ODR-Co_9_S_8_/CoO/NC heterostructure was 16.6 mF cm^−2^, suggesting a higher electrochemical surface area (ECSA) among all reference materials. However, ECSA normalized LSV curves for measuring the HER performance of the ODR-Co_9_S_8_/CoO/NC (Figure S9b) also indicate the performance excels over reference samples including ZIF-67, Co_3_S_4_/Co(OH)_2_/ZIF-67, CoO/NC, pure Co_9_S_8_, and RuO_2_/C. Again, this improved HER performance of the catalyst accustomed to oxygen vacancies generations in heterostructured ODR-Co_9_S_8_/CoO/NC. Additionally, the mass activity and TOF for ODR-Co_9_S_8_/CoO/NC are (0.98 A mg^−1^) and (1.3 × 10^−2^ mol s^−1^), respectively, which is much better than reference samples (Figure S10b and Table S3). EIS results in Figure 5e demonstrated the R_ct_ values follow the order of ODR-Co_9_S_8_/CoO/NC < Co_3_S_4_/ZIF-67 < CoO/NC < ZIF-67 ≈ pure Co_9_S_8_ which is in good accordance with the best HER performance related to heterostructure formation found in the ODR-Co_9_S_8_/CoO/NC. In Figure 5f, the catalytic performance of the ODR-Co_9_S_8_/CoO/NC almost remained unchanged after 10,000 CV cycles, proving its excellent durability in the alkaline medium for HER as well. Similar to OER, ODR-Co_9_S_8_/CoO/NC showed alike HER polarization curves even at different scan rates (1 to 100 mV s^−1^) as depicted in (Figure S11b) which indicates its stability for the active electrochemical process in alkaline solution. Post-mortem stability analysis in Figure S12 further affirmed structural solidity of catalyst even after long-term stability test. As carbon framework shield the hetero-nanoparticles which then resist agglomerations and harsh conditions during electrolysis, i.e., oxidation potentials, strong bases, and corrosion, which further confirm its excellent stability. In spite of distinctive OER performance, the as-prepared catalyst exhibited superior HER activity among previously reported co-based compounds that highlighted its potential for being a bifunctional catalyst (Table S5 of Supporting Information and Figure 5g).

## 4. Conclusion

In this work, we have synthesized a bifunctional ODR-Co_9_S_8_/CoO/NC electrocatalyst through a synergistic strategy, via annealing Co_3_S_4_/Co(OH)_2_/ZIF-67 precursor. The synergistic effects between surface-induced oxygen vacancies and heterojunction enable the ODR-Co_9_S_8_/CoO/NC to exhibit low overpotentials in both HER and OER. The current design and synthetic methodology potentially offer an alternative way to fabricate low-cost, noble-metal-free bifunctional electrocatalysts with good catalytic performance.

## Data Availability

The data is included in the main text and/or the Supplementary Materials.

## Supplementary Materials

The following are available online at https://www.mdpi.com/article/10.3390/nano11092237/s1, Figure S1: Scanning electron microscope (SEM) images of ZIF-67 (a,b) and Co_3_S_4_/Co(OH)_2_/ZIF-67 (c,d); Figure S2: (a) X-ray diffraction pattern (XRD) of ZIF-67 and Co_3_S_4_/Co(OH)_2_/ZIF-67, (b) FTIR spectrum of as-prepared Co_3_S_4_/Co(OH)_2_/ZIF-67 precursor; Figure S3: EDS analysis of ZIF-67; Figure S4: EDS analysis of Co_3_S_4_/Co(OH)_2_/ZIF-67; Figure S5: X-ray photoelectron spectroscopy (XPS) spectra of Co_3_S_4_/Co(OH)_2_/ZIF-67 precursor, (a) Co 2p, (b) S 2p, (c) C 1s, (d) O 1s; Figure S6: (a–f) HRTEM images and profile of the lattice fringes of ODR-Co_9_S_8_/CoO/NC heterostructure; Figure S7: EDX analysis of ODR-Co_9_S_8_/CoO/NC; Figure S8: CV curves of (a) ODR-Co_9_S_8_/CoO/NC, (b) Co_3_S_4_/Co(OH)_2_/ZIF-67 and (c) ZIF-67 for OER and (d) ODR-Co_9_S_8_/CoO/NC, (e) Co_3_S_4_/Co(OH)_2_/ZIF-67 and (f) ZIF-67 for HER; Figure S9: ECSA normalized LSV curves of ODR-Co_9_S_8_/CoO/NC in comparative to reference samples for (a) OER and (b) HER; Figure S10: (a) The mass activity of OER catalysts at 250 mV and (b) HER catalysts at 200 mV; Figure S11: (a,b) OER and HER polarization curves of ODR-Co_9_S_8_/CoO/NC at different scan speeds; Figure S12: The XRD spectrum of the ODR-Co_9_S_8_/CoO/NC heterostructures after electrocatalytic test; Figure S13: (a,b) OER and HER performance of Co_9_S_8_/CoO/NC-450 and Co_9_S_8_/CoO/NC-650. (c) EPR spectrum of Co_9_S_8_/CoO/NC-450 and Co_9_S_8_/CoO/NC-650; Table S1: The surface elemental composition of the as-prepared ODR-Co_9_S_8_/CoO/NC heterostructures according the XPS measurements; Table S2: Comparison of electrochemical surface area (ECSA) of ODR-Co_9_S_8_/CoO/NC and reference samples for OER and HER; Table S3: TOF of OER and HER catalysts; Table S4: Comparison of some previously reported cobalt based electrocatalysts for OER; Table S5: Comparison of some previously reported cobalt based electrocatalysts for HER.
