# Supplementary material for: In Situ Formation of Surface-Induced Oxygen Vacancies in Co9S8/CoO/NC as a Bifunctional Electrocatalyst for Improved Oxygen and Hydrogen Evolution Reactions
Source: Nanomaterials (Basel). 2021 Aug 30;11(9):2237. doi: 10.3390/nano11092237 (PMC8471348; doi:10.3390/nano11092237)
Supplement: Supplementary file 1 [file nanomaterials-11-02237-s001.zip › nanomaterials-1341534-supplementary.pdf]

Supporting Information

In-situ Formation of Surface-induced Oxygen Vacancies in Co_9S_8/CoO/NC as a Bifunctional Electrocatalyst for Improved Oxygen and Hydrogen Evolution Reactions

Khalil ur Rehman, Shaista Airam, Xiangyun Lin, Jian Gao, Qiang Guo and Zhipan Zhang

Calculations for mass activity (MA), turn over frequency (TOF) and current density normalized by electrochemical active surface area.

The mass activity (MA) is calculated according to Eqn. S1:

$$MA = J/m \qquad \text{Eqn. S1}$$

where J (mA cm^{-2}) is the current density and m for mass of electrocatalytic active sites deposited onto the GC electrode.

The turn over frequency (TOF) of the catalyst for OER and HER can be estimated by Eqn. S2 and S3.[S1]

$$TOF_{OER} = I/4Fn \qquad \text{Eqn. S2}$$

$$TOF_{HER} = I/2Fn \qquad \text{Eqn. S3}$$

Where I is the current (A) according to the linear sweep measurement, F is the Faraday constant (F = 96485 C mol^{-1}), n is the number of moles of surface atoms, which is estimated according to the equation S4. The factor 1/2 and 1/4 is based on consideration that two and four electrons are required to form one hydrogen and oxygen molecule.

$$n = [m_x/100M_s]\times(\text{surface atoms\%}) \qquad \text{Eqn. S4}$$

where m is the weight (g) of the catalyst that loaded on GC electrode, x is weight proportion of the metal obtained from EDS, and M_s is the molar weight of the metal. While surface atoms% is estimated by dividing the thickness of the wall of the hollow dodecahedral structure (30 nm)

with the thickness of the single layer of Co_9S_8 or CoO (0.426 nm). Here the estimated surface atoms% is 1.4% while for reference samples the same percentage has used.

The current density normalized by electrochemical active surface area (J_{ECSA}) is calculated by

$$J_{ECSA} = I/_{ECSA} \qquad \text{Eqn. S5}$$

where I is the catalytic current (mA) and $ECSA$ is the electrochemically active surface area of the catalyst which is calculated according to Eqn. S6

$$ECSA = C_{dl}/C_S \qquad \text{Eqn. S6}$$

Where C_S is the specific capacitance, and its value was taken to be 40 μF cm^{-2}.[S2] Noted that because it is difficult to determine the catalytic electrochemical surface area significantly in-real [S3,S4] a Cs value of 40 μF cm^{-2} was thus adopted according to prior reported Co-based catalysts [S5] while the value of C_{dl} was calculated from cyclic voltammetry (CV) tests at different scan rates of 20-100 mV s^{-1} in a narrow potential range selected between 1.02-1.12 V vs. RHE. Half of the linear slope gave the value of C_{dl} and obtained by plotting Δj versus different scan rates.

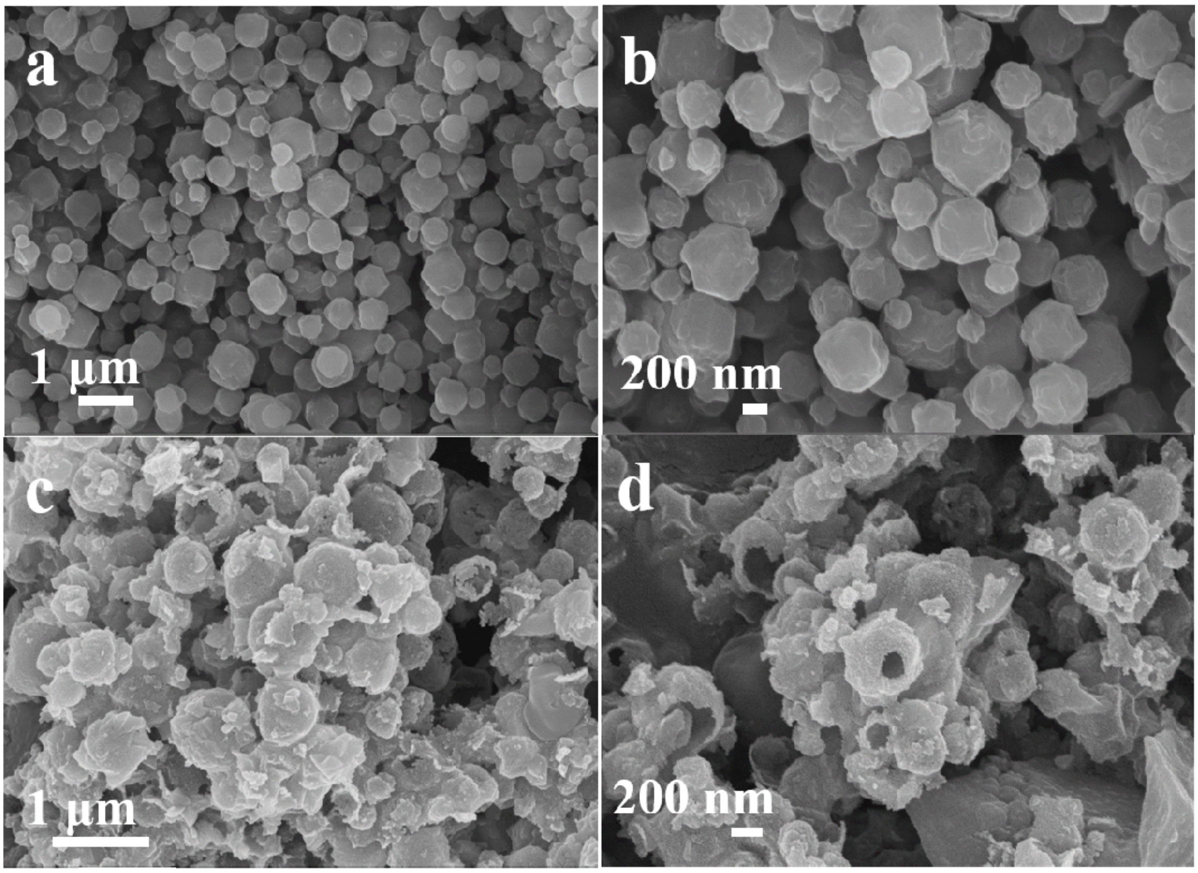

Figure S1 Scanning electron microscope (SEM) images of ZIF-67 **(a, b)** and $Co_3S_4/Co(OH)_2$/ZIF-67 (**c, d**).

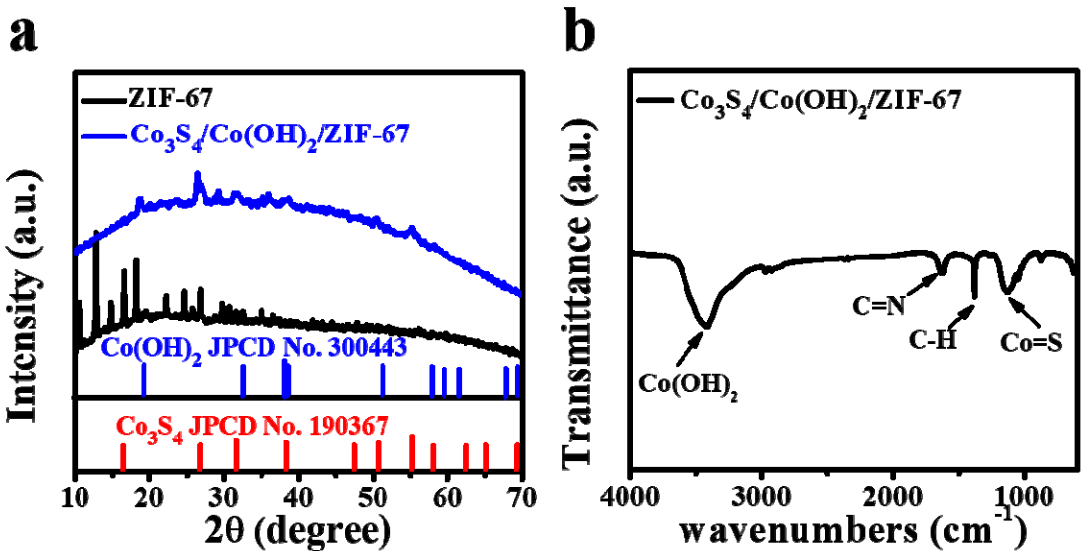

Figure S2 (a) X-ray diffraction pattern (XRD) of ZIF-67 and $Co_3S_4/Co(OH)_2$/ZIF-67, **(b)** FTIR spectrum of as-prepared $Co_3S_4/Co(OH)_2$/ZIF-67 precursor.

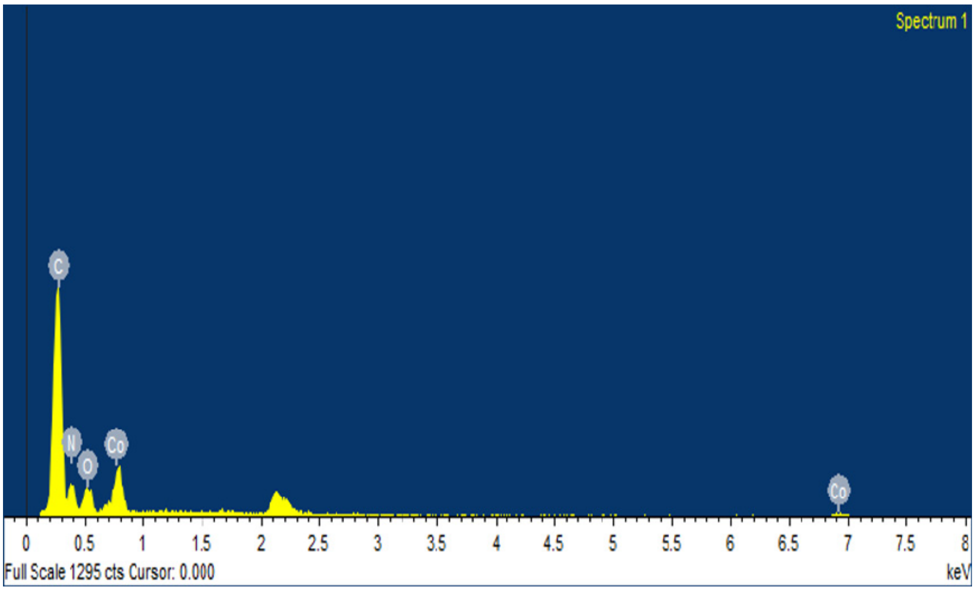

Figure S3 EDS analysis of ZIF-67.

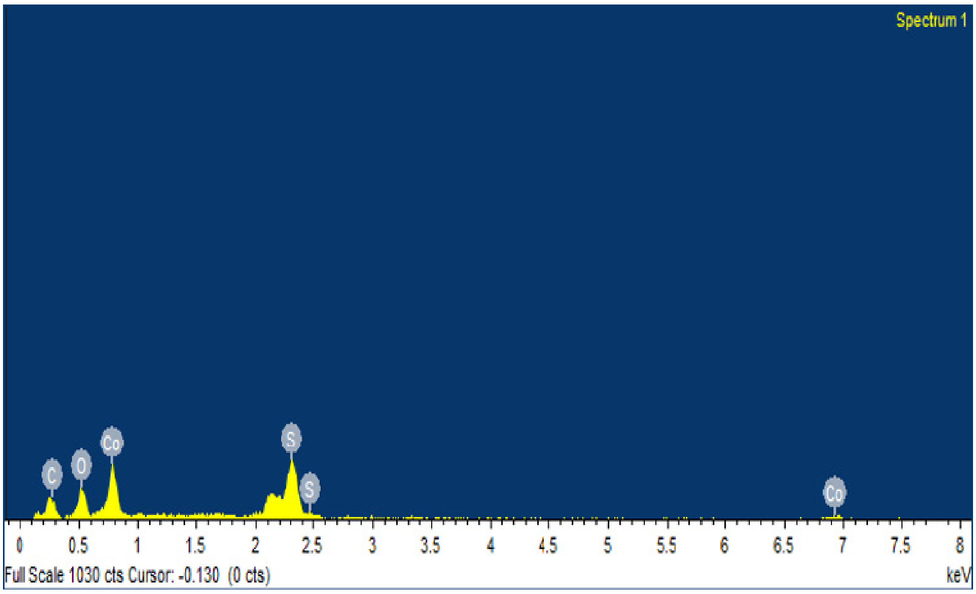

Figure S4 EDS analysis of $Co_3S_4/Co(OH)_2$/ZIF-67.

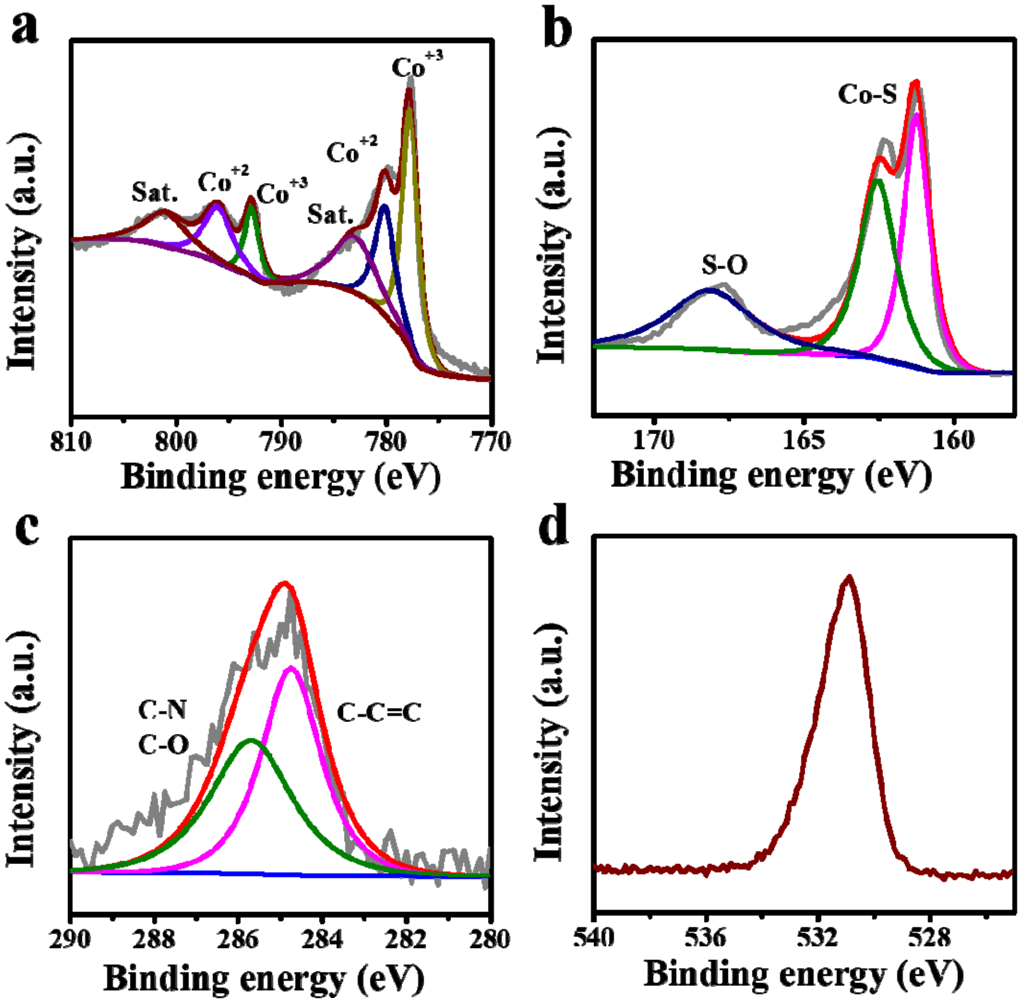

Figure S5 X-ray photoelectron spectroscopy (XPS) spectra of $Co_3S_4/Co(OH)_2$/ZIF-67 precursor, **(a)** Co 2p, **(b)** S 2p, **(c)** C 1s, **(d)** O 1s.

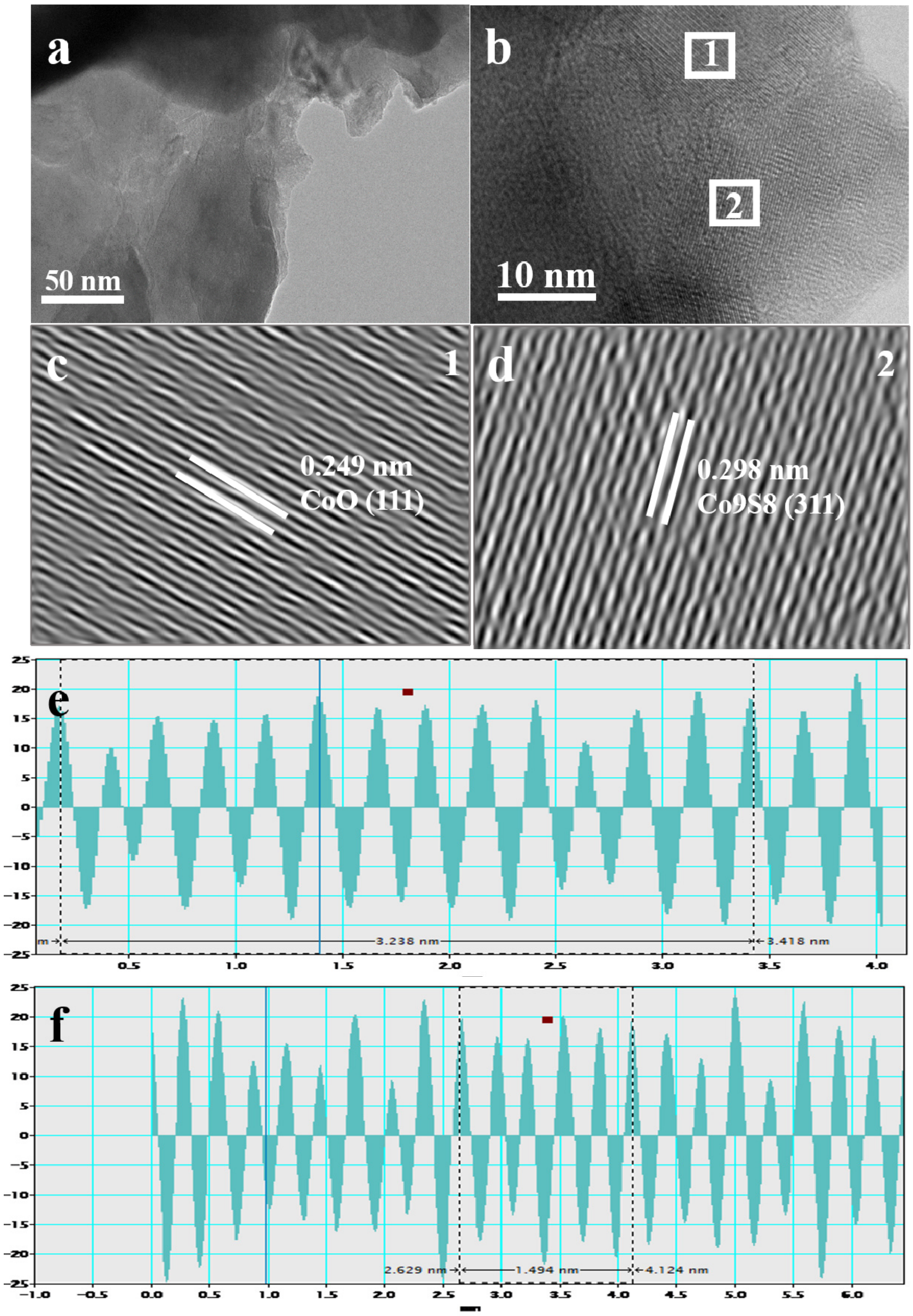

Figure S6 (a-f) HRTEM images and profile of the lattice fringes of ODR-Co_9S_8/CoO/NC heterostructure.

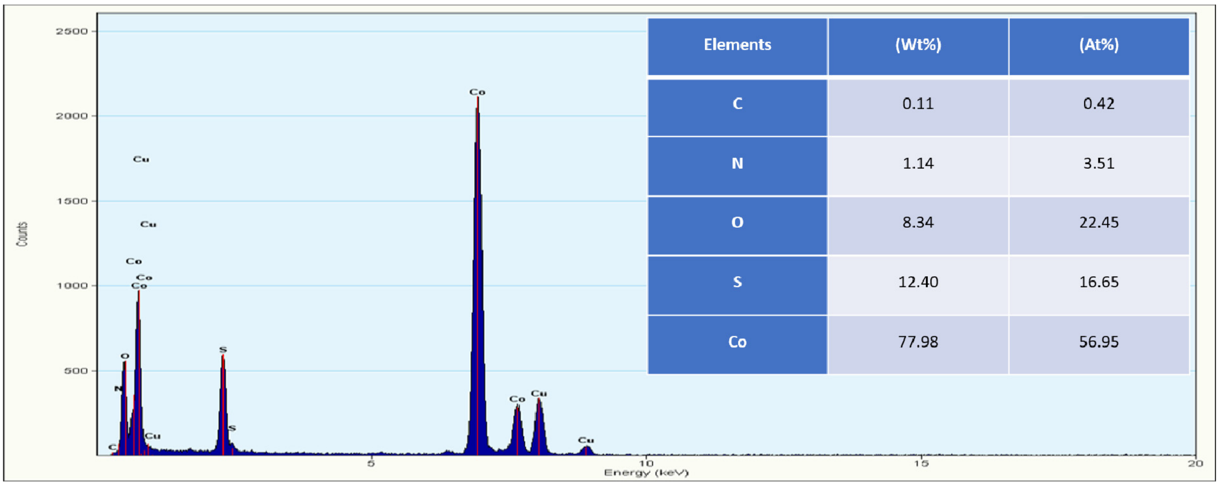

Elements	(Wt%)	(At%)
C	0.11	0.42
N	1.14	3.51
O	8.34	22.45
S	12.40	16.65
Co	77.98	56.95

Figure S7 EDX analysis of ODR-Co_9S_8/CoO/NC.

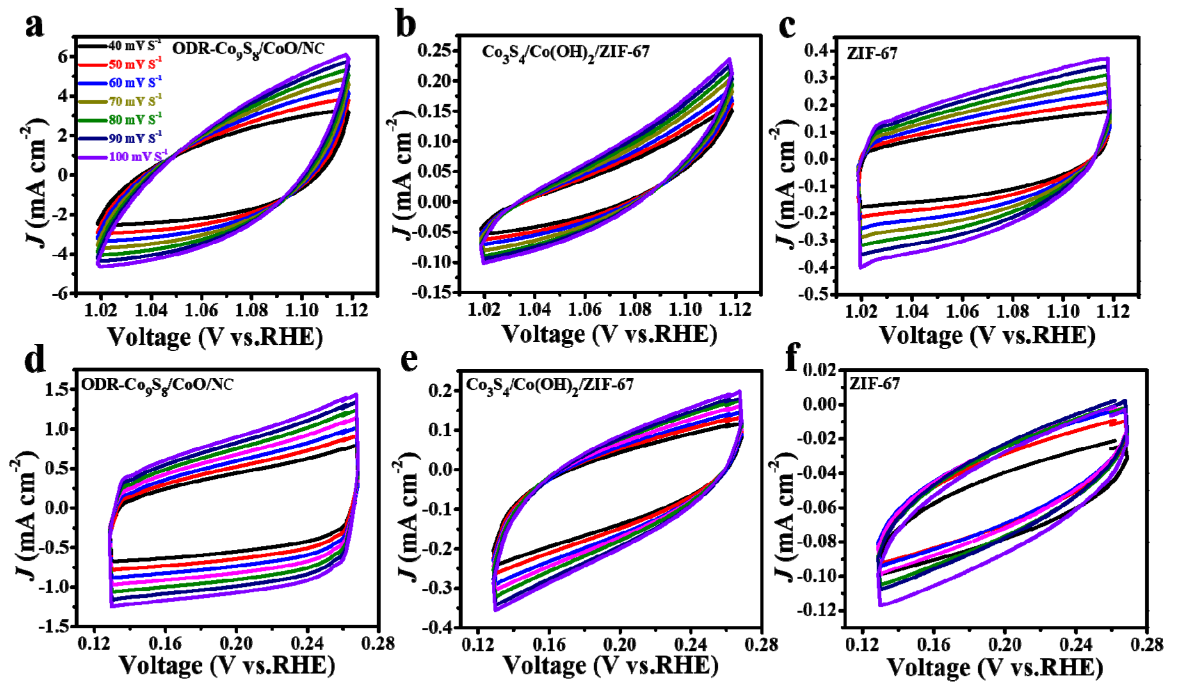

Figure S8 CV curves of **(a)** ODR-Co_9S_8/CoO/NC, **(b)** $Co_3S_4/Co(OH)_2$/ZIF-67 and **(c)** ZIF-67 for OER and **(d)** ODR-Co_9S_8/CoO/NC, **(e)** $Co_3S_4/Co(OH)_2$/ZIF-67 and **(f)** ZIF-67 for HER.

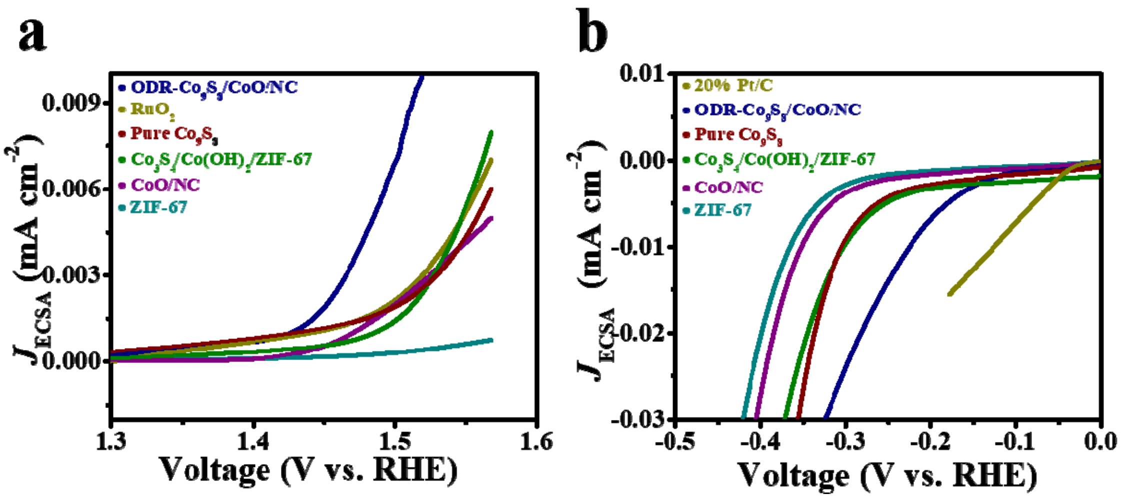

Figure S9 ECSA normalized LSV curves of ODR-Co_9S_8/CoO/NC in comparative to reference samples for **(a)** OER and **(b)** HER.

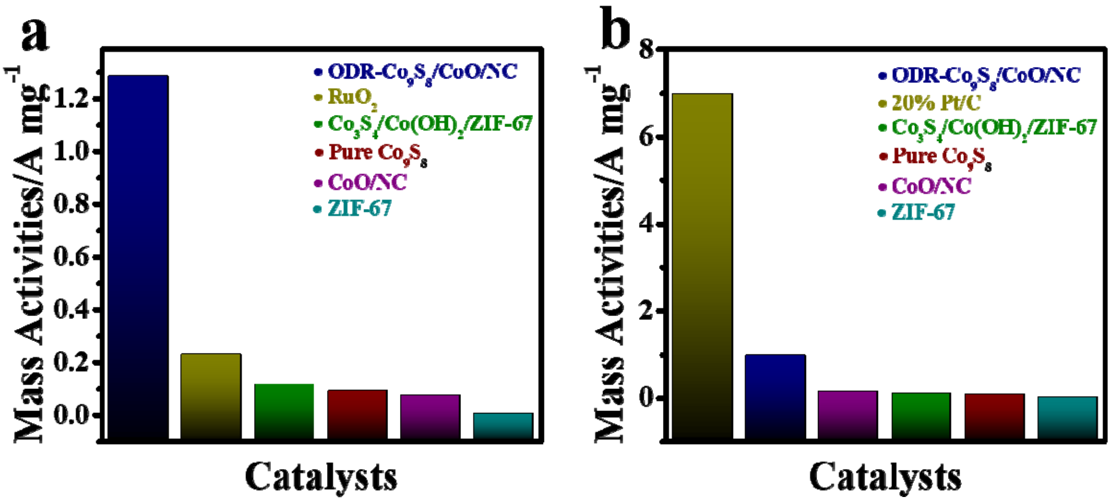

Figure S10 (a) The mass activity of OER catalysts at 250 mV and **(b)** HER catalysts at 200 mV.

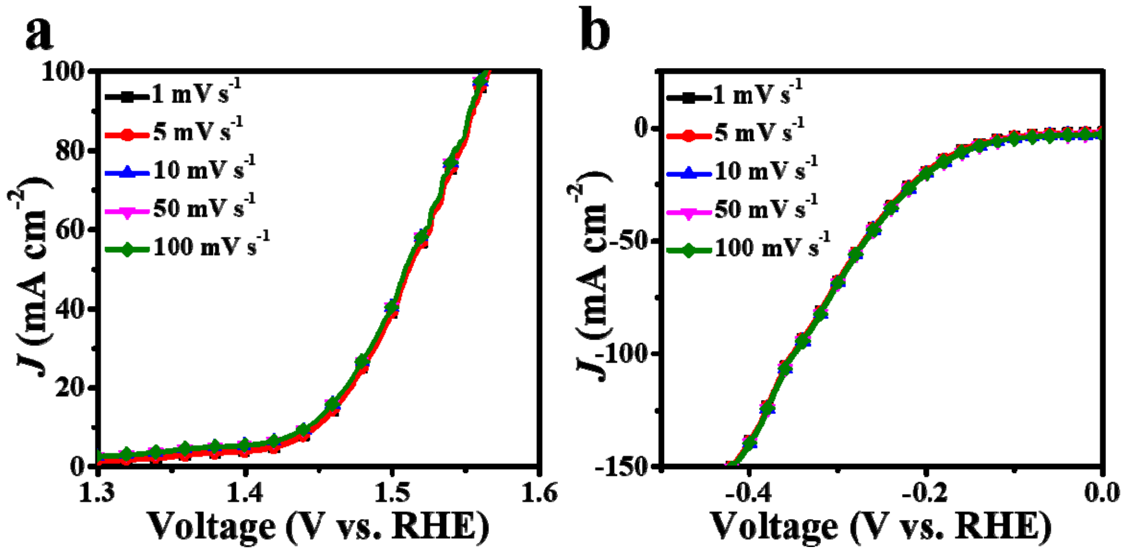

Figure S11 (a-b) OER and HER polarization curves of ODR-Co_9S_8/CoO/NC at different scan speeds.

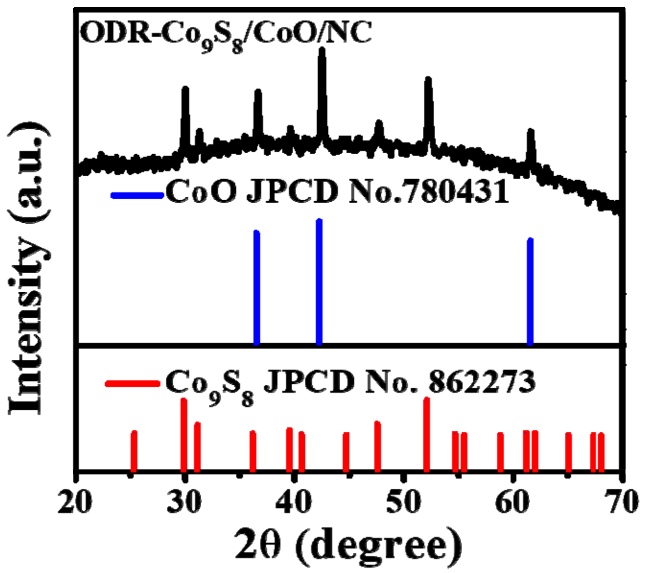

Figure S12 The XRD spectrum of the ODR-Co_9S_8/CoO/NC heterostructures after electrocatalytic test.

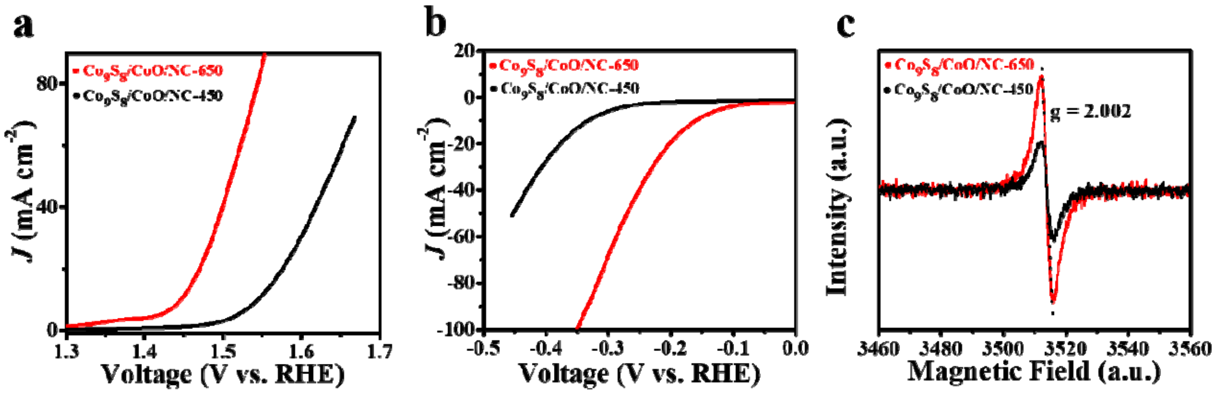

Figure S13 (a, b) OER and HER performance of Co_9S_8/CoO/NC-450 and Co_9S_8/CoO/NC-650. **(c)** EPR spectrum of Co_9S_8/CoO/NC-450 and Co_9S_8/CoO/NC-650.

Table S1 The surface elemental composition of the as-prepared ODR-Co_9S_8/CoO/NC heterostructures according the XPS measurements.

Samples	Co/XPS (At. %)	N/XPS (At. %)	C/XPS (At. %)	S/XPS (At. %)	O/XPS (At. %)
ODR-Co_9S_8/CoO/NC	14.99	1.96	14.79	14.49	53.77

Table S2 Comparison of electrochemical surface area (ECSA) of ODR-Co_9S_8/CoO/NC and reference samples for OER and HER.

**Catalysts**	**C_{dl} (mF cm^{-2}) (OER)**	**ECSA (cm^2) (OER)**	**C_{dl} (mF cm^{-2}) (HER)**	**ECSA (cm^2) (HER)**
ODR-Co_9S_8/CoO/NC	39.4	985	16.6	415
Co_3S_4/Co(OH)$_2$/ZIF-67	4.1	102.5	1.74	43.5
Pure Co_9S_8	6	150	1.6	40
CoO/NC	2.8	70	0.8	20
ZIF-67	1	25	0.05	1.25

Table S3 TOF of OER and HER catalysts.

**Catalyst**	**TOF (OER)** (mol s^{-1})	**TOF (HER)** (mol s^{-1})
ODR-Co_9S_8/CoO/NC	9.3×10^{-3}	1.3×10^{-2}
Co_3S_4/$Co(OH)_2$/ZIF-67	1.6×10^{-3}	2.1×10^{-3}
Pure Co_9S_8	2.4×10^{-3}	1.7×10^{-3}
CoO/NC	7×10^{-4}	1×10^{-3}
ZIF-67	1.3×10^{-4}	3.9×10^{-4}

Table S4 Comparison of some previously reported cobalt based electrocatalysts for OER.

Catalyst	Mass loading (mg cm^{-2})	Electrolyte	$\eta_{(J=10\ mA\ cm^{-2})}$ (mV)	Tafel slope (mV dec^{-1})	Reference
CoO hexagrams	0.27	1 M KOH	269	64.4	[S6]
CoO nanorods	0.4	1 M KOH	330	44	[S7]
CoOx@NC nanoarrays	0.45	0.1 M KOH	348	N/A	[S8]
CoO/C nanocrystals	0.31	0.1 M KOH	362	45.2	[S9]
N–CoO nanowires	N/A	1M KOH	319	74	[S10]
CoO/NF	N/A	1M KOH	307	72	[S11]
Co–CoO/BC yolk-shell	N/A	1M KOH	300	73.3	[S12]
Co_9S_8 hollow microplates	0.37	1M KOH	278	53	[S13]
Co_9S_8/CS	0.15	0.1 M KOH	370	98	[S14]
Co/S/N-800	0.1	0.1 M KOH	361	74	[S15]
Co_9S_8/NSCNFs	0.21	1 M KOH	302	54	[S16]
Co_9S_8@NOSC	0.28	1 M KOH	340	68	[S17]
Co/Co_9S_8@SNGS	0.30	0.1 M KOH	290	80.2	[S18]
Co_3O_4-CoO heterostructure	N/A	1 M KOH	270	49	[S19]
Co_9S_8/Co_3O_4 heterostructure	N/A	1 M KOH	250	73.5	[S20]
Co_9S_8/N,S-rGO	N/A	1 M KOH	266	75.5	[S21]
ODR-Co_9S_8/CoO/NC	0.28	0.1 M KOH	217	70	**This work**

Table S5 Comparison of some previously reported cobalt based electrocatalysts for HER.

**Catalyst**	**Mass loading (mg cm^{-2})**	**Electrolyte**	$\eta_{(J=10\ mA\ cm^{-2})}$ **(mV)**	**Tafel slope (mV dec^{-1})**	**Reference**
CoO/NF	N/A	1 M KOH	224	72	[S11]
CoO/N-S-UPCNPs	0.28	1 M KOH	110	94	[S22]
C@CoO/CC	N/A	1 M KOH	120	129	[S23]
CoOx@CN	0.12	1 M KOH	232	N/A	[S24]
Co@CoO/NG	2	1 M KOH	112	119	[S25]
Co/CoO	N/A	1 M KOH	160	68.1	[S26]
Co_9S_8/Co_3O_4	N/A	1 M KOH	250	73.5	[S20]
Co/Co_9S_8@NSOC	0.64	1 M KOH	216	149	[S27]
Co_9S_8/CoNCNT	0.56	1 M KOH	196	84.9	[S28]
Co_9S_8/N,S-rGO	0.37	1 M KOH	334.2	118	[S21]
Co_9S_8@N-S-HPC	0.26	1 M KOH	173	78	[S29]
N,S- Co_9S_8 NPS/MC	N/A	1 M KOH	196	75.5	[S30]
CoSA+Co_9S_8/HCNT	0.97	1 M KOH	250	101	[S31]
Co_9S_8@C	0.3	1 M KOH	250	NA	[S32]
ODR-Co_9S_8/CoO/NC	0.28	0.1 M KOH	160	90	**This work**

References to supporting information

[S1]. Cheng, Z.; Fu, Q.; Han, Q.; Xiao, Y.; Liang, Y.; Zhao, Y.; Qu, L. A Type of 1 nm Molybdenum Carbide Confined within Carbon Nanomesh as Highly Efficient Bifunctional Electrocatalyst. *Adv. Funct. Mater.* 2018, *28*, 1705967, doi:https://doi.org/10.1002/adfm.201705967.

[S2]. McCrory, C.C.L.; Jung, S.; Peters, J.C.; Jaramillo, T.F. Benchmarking Heterogeneous Electrocatalysts for the Oxygen Evolution Reaction. *J. Am. Chem. Soc.* 2013, *135*, 16977–16987, doi:10.1021/ja407115p.

[S3]. Sun, S.; Li, H.; Xu, Z.J. Impact of Surface Area in Evaluation of Catalyst Activity. *Joule* 2018, *2*, 1024–1027, doi:https://doi.org/10.1016/j.joule.2018.05.003.

[S4]. Zheng, Y.-R.; Wu, P.; Gao, M.-R.; Zhang, X.-L.; Gao, F.-Y.; Ju, H.-X.; Wu, R.; Gao, Q.; You, R.; Huang, W.-X.; et al. Doping-induced structural phase transition in cobalt diselenide enables enhanced hydrogen evolution catalysis. *Nat. Commun.* 2018, *9*, 2533, doi:10.1038/s41467-018-04954-7.

[S5]. Zhang, Y.; Shao, Q.; Pi, Y.; Guo, J.; Huang, X. A Cost-Efficient Bifunctional Ultrathin Nanosheets Array for Electrochemical Overall Water Splitting. *Small* 2017, *13*, 1700355, doi:https://doi.org/10.1002/smll.201700355.

[S6]. Liang, Z.; Huang, Z.; Yuan, H.; Yang, Z.; Zhang, C.; Xu, Y.; Zhang, W.; Zheng, H.; Cao, R. Quasi-single-crystalline CoO hexagrams with abundant defects for highly efficient electrocatalytic water oxidation. *Chem. Sci.* 2018, *9*, 6961–6968, doi:10.1039/C8SC02294A.

[S7]. Ling, T.; Yan, D.-Y.; Jiao, Y.; Wang, H.; Zheng, Y.; Zheng, X.; Mao, J.; Du, X.-W.; Hu, Z.; Jaroniec, M.; et al. Engineering surface atomic structure of single-crystal cobalt (II) oxide nanorods for superior electrocatalysis. *Nat. Commun.* 2016, *7*, 12876, doi:10.1038/ncomms12876.

[S8]. Hao, Y.; Xu, Y.; Han, N.; Liu, J.; Sun, X. Boosting the bifunctional electrocatalytic oxygen activities of CoOx nanoarrays with a porous N-doped carbon coating and their application in Zn–air batteries. *J. Mater. Chem. A* 2017, *5*, 17804–17810,

doi:10.1039/C7TA03996D.

[S9]. Kim, H.; Kim, Y.; Noh, Y.; Lee, S.; Sung, J.; Kim, W.B. Thermally Converted CoO Nanoparticles Embedded into N-Doped Carbon Layers as Highly Efficient Bifunctional Electrocatalysts for Oxygen Reduction and Oxygen Evolution Reactions. *ChemCatChem* 2017, *9*, 1503–1510, doi:10.1002/cctc.201601705.

[S10]. Zhang, K.; Xia, X.; Deng, S.; Xie, D.; Lu, Y.; Wang, Y.; Wu, J.; Wang, X.; Tu, J. N-doped CoO nanowire arrays as efficient electrocatalysts for oxygen evolution reaction. *J. Energy Chem.* 2019, *37*, 13–17, doi:https://doi.org/10.1016/j.jechem.2018.11.013.

[S11]. Zhu, S.; Lei, J.; Zhang, L.; He, J. CoO/NF nanowires promote hydrogen and oxygen production for overall water splitting in alkaline media. *Int. J. Hydrogen Energy* 2020, *45*, 8031–8040, doi:https://doi.org/10.1016/j.ijhydene.2020.01.085.

[S12]. Yang, M.; Wu, D.; Cheng, D. Biomass-derived porous carbon supported CoCoO yolk-shell nanoparticles as enhanced multifunctional electrocatalysts. *Int. J. Hydrogen Energy* 2019, *44*, 6525–6534, doi:https://doi.org/10.1016/j.ijhydene.2019.01.155.

[S13]. Liu, H.; Ma, F.-X.; Xu, C.-Y.; Yang, L.; Du, Y.; Wang, P.-P.; Yang, S.; Zhen, L. Sulfurizing-Induced Hollowing of Co9S8 Microplates with Nanosheet Units for Highly Efficient Water Oxidation. *ACS Appl. Mater. Interfaces* 2017, *9*, 11634–11641, doi:10.1021/acsami.7b00899.

[S14]. Li, W.; Li, Y.; Wang, H.; Cao, Y.; Yu, H.; Peng, F. Co9S8-porous carbon spheres as bifunctional electrocatalysts with high activity and stability for oxygen reduction and evolution reactions. *Electrochim. Acta* 2018, *265*, 32–40, doi:https://doi.org/10.1016/j.electacta.2018.01.095.

[S15]. Jia, N.; Liu, J.; Gao, Y.; Chen, P.; Chen, X.; An, Z.; Li, X.; Chen, Y. Graphene-Encapsulated Co_9S_8 Nanoparticles on N,S-Codoped Carbon Nanotubes: An Efficient Bifunctional Oxygen Electrocatalyst. *ChemSusChem* 2019, *12*, 3390–3400, doi:10.1002/cssc.201900383.

[S16]. Wu, L.-L.; Wang, Q.-S.; Li, J.; Long, Y.; Liu, Y.; Song, S.-Y.; Zhang, H.-J. Co_9S_8 Nanoparticles-Embedded N/S-Codoped Carbon Nanofibers Derived from Metal–Organic

Framework-Wrapped CdS Nanowires for Efficient Oxygen Evolution Reaction. *Small* 2018, *14*, 1704035, doi:10.1002/smll.201704035.

[S17]. Huang, S.; Meng, Y.; He, S.; Goswami, A.; Wu, Q.; Li, J.; Tong, S.; Asefa, T.; Wu, M. N-, O-, and S-Tridoped Carbon-Encapsulated Co_9S_8 Nanomaterials: Efficient Bifunctional Electrocatalysts for Overall Water Splitting. *Adv. Funct. Mater.* 2017, *27*, 1606585, doi:10.1002/adfm.201606585.

[S18]. Zhang, X.; Liu, S.; Zang, Y.; Liu, R.; Liu, G.; Wang, G.; Zhang, Y.; Zhang, H.; Zhao, H. Co/Co_9S_8@S,N-doped porous graphene sheets derived from S, N dual organic ligands assembled Co-MOFs as superior electrocatalysts for full water splitting in alkaline media. *Nano Energy* 2016, *30*, 93–102, doi:https://doi.org/10.1016/j.nanoen.2016.09.040.

[S19]. Qi, J.; Yan, Y.; Liu, T.; Zhou, X.; Cao, J.; Feng, J. Plasma-induced surface reorganization of porous Co_3O_4-CoO heterostructured nanosheets for electrocatalytic water oxidation. *J. Colloid Interface Sci.* 2020, *565*, 400–404, doi:https://doi.org/10.1016/j.jcis.2020.01.045.

[S20]. Peng, D.; Zhang, B.; Wu, J.; Huang, K.; Cao, X.; Lu, Y.; Zhang, Y.; Li, C.; Huang, Y. Growth of Lattice Coherent Co_9S_8/Co_3O_4 Nano-Heterostructure for Maximizing the Catalysis of Co-Based Composites. *ChemCatChem* 2020, *12*, 2431–2435, doi:10.1002/cctc.202000044.

[S21]. Liu, H.; Xu, C.-Y.; Du, Y.; Ma, F.-X.; Li, Y.; Yu, J.; Zhen, L. Ultrathin Co_9S_8 nanosheets vertically aligned on N,S/rGO for low voltage electrolytic water in alkaline media. *Sci. Rep.* 2019, *9*, 1951, doi:10.1038/s41598-018-35831-4.

[S22]. Chao, S.; Wu, H.; Xia, Q.; Wang, G. Ultrathin Two-Dimensional Metal-Organic-Framework-Derived CoO/Nitrogen and Sulfur Co-doped Ultrathin Porous Carbon Nanoplates for Highly Efficient Water Electrolysis. *ChemElectroChem* 2019, *6*, 3940–3948, doi:10.1002/celc.201900939.

[S23]. Jin, W.; Guo, X.; Zhang, J.; Zheng, L.; Liu, F.; Hu, Y.; Mao, J.; Liu, H.; Xue, Y.; Tang, C. Ultrathin carbon coated CoO nanosheet arrays as efficient electrocatalysts for the hydrogen evolution reaction. *Catal. Sci. Technol.* 2019, *9*, 6957–6964, doi:10.1039/C9CY01645G.

[S24]. Jin, H.; Wang, J.; Su, D.; Wei, Z.; Pang, Z.; Wang, Y. In situ Cobalt–Cobalt Oxide/N-Doped Carbon Hybrids As Superior Bifunctional Electrocatalysts for Hydrogen and Oxygen Evolution. *J. Am. Chem. Soc.* 2015, *137*, 2688–2694, doi:10.1021/ja5127165.

[S25]. Zhang, S.; Yu, X.; Yan, F.; Li, C.; Zhang, X.; Chen, Y. N-Doped graphene-supported Co@CoO core–shell nanoparticles as high-performance bifunctional electrocatalysts for overall water splitting. *J. Mater. Chem. A* 2016, *4*, 12046–12053, doi:10.1039/C6TA04365H.

[S26]. Cao, J.; Chen, X.; Li, H.; Pu, J.; Liu, L.; Ma, L.; Zhou, K.; Zhang, Z.; Wei, Q.; Luo, F. A Co/CoO hybrid rooted on carbon cloth as an efficient electrocatalyst for the hydrogen evolution reaction in alkaline solution. *Sustain. Energy Fuels* 2020, *4*, 1924–1932, doi:10.1039/C9SE01128E.

[S27]. Du, J.; Wang, R.; Lv, Y.-R.; Wei, Y.-L.; Zang, S.-Q. One-step MOF-derived Co/Co_9S_8 nanoparticles embedded in nitrogen, sulfur and oxygen ternary-doped porous carbon: an efficient electrocatalyst for overall water splitting. *Chem. Commun.* 2019, *55*, 3203–3206, doi:10.1039/C9CC00196D.

[S28]. Liang, D.; Mao, J.; Liu, P.; Yan, J.; Song, W. In-situ growth of NCNT and encapsulation of Co_9S_8/Co as a sustainable multifunctional electrocatalyst. *J. Colloid Interface Sci.* 2019, *557*, 291–300, doi:https://doi.org/10.1016/j.jcis.2019.09.018.

[S29]. Zhang, S.; Zhai, D.; Sun, T.; Han, A.; Zhai, Y.; Cheong, W.-C.; Liu, Y.; Su, C.; Wang, D.; Li, Y. In situ embedding Co_9S_8 into nitrogen and sulfur codoped hollow porous carbon as a bifunctional electrocatalyst for oxygen reduction and hydrogen evolution reactions. *Appl. Catal. B Environ.* 2019, *254*, 186–193, doi:https://doi.org/10.1016/j.apcatb.2019.04.096.

[S30]. Yu, H.; Zhang, W.; Miao, S.; Du, Y.; Huang, Y.; Tang, D.; Qiao, Z.-A.; Wang, J.; Zhao, Z. Synthesis of Co_9S_8 nanoparticle embedded, N, S Co-doped mesoporous carbon with salts as templates for electrocatalytic hydrogen evolution. *Microporous Mesoporous Mater.* 2020, *302*, 110235, doi:https://doi.org/10.1016/j.micromeso.2020.110235.

[S31]. Li, Y.; Cao, R.; Li, L.; Tang, X.; Chu, T.; Huang, B.; Yuan, K.; Chen, Y. Simultaneously

Integrating Single Atomic Cobalt Sites and Co_9S_8 Nanoparticles into Hollow Carbon Nanotubes as Trifunctional Electrocatalysts for Zn–Air Batteries to Drive Water Splitting. *Small* 2020, *16*, 1906735, doi:10.1002/smll.201906735.

[S32]. Feng, L.-L.; Li, G.-D.; Liu, Y.; Wu, Y.; Chen, H.; Wang, Y.; Zou, Y.-C.; Wang, D.; Zou, X. Carbon-Armored Co_9S_8 Nanoparticles as All-pH Efficient and Durable H_2-Evolving Electrocatalysts. *ACS Appl. Mater. Interfaces* 2015, *7*, 980–988, doi:10.1021/am507811a.
